# A Fitness-Fatigue Model of Performance in Peripheral Artery Disease: Predicted and Measured Effects of a Pain-Free Exercise Program

**DOI:** 10.3390/jpm12030397

**Published:** 2022-03-04

**Authors:** Nicola Lamberti, Giovanni Piva, Federico Businaro, Lorenzo Caruso, Anna Crepaldi, Pablo Jesùs Lòpez-Soto, Fabio Manfredini

**Affiliations:** 1 Department of Neuroscience and Rehabilitation, University of Ferrara, Via Luigi Borsari 46, 44121 Ferrara, Italy; nicola.lamberti@unife.it (N.L.); federico.businaro@hotmail.it (F.B.); anna.crepaldi@edu.unife.it (A.C.); 2 PhD Program in Environmental Sustainability and Wellbeing, Department of Humanistic Studies, University of Ferrara, 44121 Ferrara, Italy; giovanni.piva@unife.it; 3 Department of Environmental Sciences and Prevention, University of Ferrara, 44121 Ferrara, Italy; lorenzo.caruso@unife.it; 4 Department of Nursing, Instituto Maimónides de Investigación Biomédica de Córdoba, 14005 Córdoba, Spain; n82losop@uco.es; 5 Department of Nursing, Universidad de Córdoba, 14004 Córdoba, Spain; 6 Department of Rehabilitation Medicine, University Hospital of Ferrara, 44124 Ferrara, Italy

**Keywords:** exercise therapy, rehabilitation, peripheral artery disease, impulse-response, gender differences, training

## Abstract

Banister impulse-response (IR) model estimates the performance in response to the training impulses (TRIMPs). In 100 patients with peripheral artery disease (PAD), we tested by an IR model the predictability of the effects of a 6-month structured home-based exercise program. The daily TRIMPs obtained from prescribed walking speed, relative intensity and time of exercise determined the fitness-fatigue components of performance. The estimated performance values, calculated from the baseline 6-min and pain-free walking distance (6MWD and PFWD, respectively) were compared with values measured at visits through regression models. Interval pain-free walking at controlled speed prescribed during circa-monthly hospital visits (5 ± 1) was safely performed at home with good adherence (92% of scheduled sessions, 144 ± 25 km walked in 50 ± 8 training hours). The mean TRIMP rose throughout the program from 276 to 601 a.u. The measured 6MWD and PFWD values increased (+33 m and +121 m, respectively) showing a good fit with those estimated by the IR model (6MWD: R^2^ 0.81; PFWD: R^2^ 0.68) and very good correspondence (correlation coefficients: 0.91 to 0.95), without sex differences. The decay of performance without training was estimated at 18 ± 3 weeks. In PAD, an IR model predicted the walking performance following a pain-free exercise program. IR models may contribute to design and verify personalized training programs.

## 1. Introduction

Exercise is an organized series of stressors that may improve physical performance according to the quality and organization of the training impulses and the body response [1,2].

The structured organization of training variables [sets, repetitions, load], the so-called periodization, enables the positive adaptation of stressors and the optimization of the balance between the negative and positive effects of training or fatigue and fitness [1,3,4]. Inspired by the interesting objective to foresee these effects, the research of several authors involved in exercise physiology, as magisterially reviewed by Clarke et al. [1], has tried to develop mathematical models to predetermine/calculate the adaptive response to training stressors [1,5,6].

This approach, primarily of interest in sports to foster the performance and health of athletes [1,7,8,9,10,11], was also considered of interest for rehabilitation [4] or for subjects with chronic diseases, such as patients undergoing cardiac rehabilitation [12,13,14], to design effective and sustainable programs. Nevertheless, this approach has been poorly exploited in general [1,12], particularly for patients with peripheral artery disease (PAD), where exercise is a cornerstone of treatment in the intermediate stages when reduced oxygen delivery affects walking capacity, quality of life and cardiovascular health [15]. However, for PAD patients, walking represents both a noninvasive option to improve mobility [15] and a stressful factor due to muscle pain and psychological-related issues [16,17,18]. Moreover, repeated bouts of exercise at pain tolerance, such as those recommended during supervised treadmill training or for home training [15,18,19], may induce muscle damage [20,21] without hemodynamic improvements [22] and with variable effects according to the severity of disease or the characteristics of the patients [15,21,23]. Unlike this approach, a more aerobic program based on short pain-free walking periods was proposed into an original test-in train-out (TiTo) program prescribed at the hospital and performed at home at a controlled speed [24,25]. This program showed good adherence and was associated with functional and hemodynamic adaptations over the course of the rehabilitative phases [24,25,26,27,28]. We hypothesize that a proper combination of training variables structured according to the exercise principles aimed at attaining aerobic adaptations should evoke theoretically predictable progressive changes in walking performance in patients with PAD with equal response independently of sex.

This study aims to verify whether a mathematical impulse-response model of athletic training can be successfully applied in a real-world PAD population with intermittent claudication by determining whether the predicted effects related to the training stimuli correspond to the performance obtained during a rehabilitative exercise program.

## 2. Materials and Methods

From July 2017 to December 2018, consecutive PAD patients addressed to the vascular rehabilitation program were evaluated for eligibility. Male and female patients with PAD at Rutherford’s stages 1–3 were enrolled in the rehabilitation program. This observational study was approved by the CE-AVEC Ethics Committee (number 277/2019).

### 2.1. Performance Assessment

The 6-min walking test (6MWT), a test validated in people with PAD [29], was employed to assess walking performance at baseline (T0).

Patients were instructed to walk as far as possible for 6 min, with the possibility to rest and restart in case of impossibility to continue walking. The distance at the onset of symptoms referred (pain-free walking distance, PFWD) and the total distance covered (6-min walking distance, 6MWD) were collected.

The individual habitual speed was derived by observing the patient and counting the number of steps during the first minute of the test.

Performance assessment was repeated during all the following hospital visits, scheduled at 5 ± 1 weeks (T1), 12 ± 2 weeks (T2), 20 ± 2 weeks (T3) and 28 ± 3 weeks (T4).

### 2.2. Training Program

During serial circa-monthly visits, patients received at the hospital the first and the updated prescription of the Ti-To program to be performed at home. The 6-month program is composed of two daily 10-min sessions of intermittent walking (1:1 walk: rest ratio) at a prescribed speed. The speed converted into steps or beats/min is respected at home by walking in rhythm with a metronome. The speed was initially set at approximately 60% of the individual habitual walking speed and then increased each week by 3% until reaching 90–100%. A diary was provided to each patient to record the exercise session execution, and any possible symptoms which occurred. More details on the exercise program are reported elsewhere [24,25].

Possible changes in working time and volume—or total number of steps in the session—may also be settled, also by phone, in relation to intercurrent diseases or personal problems.

### 2.3. Modeling the Performance

The IR model used in this study for performance modeling is based on the principles determined by Banister et al. [5]. This system analyzes the physiological adaptations following training by determining into a mathematical relationship the two components of fitness and fatigue, with performance corresponding to the difference between them. The fitness and fatigue components linearly respond to the training load and are quantified as training impulses (TRIMPs).

### 2.4. Training Quantification

According to the systems model reported by Banister et al. (1975) [5,10], the daily training quantity w(t), or TRIMP, was calculated as the product of three parameters:

- Intensity: the ratio between the training speed and the habitual walking speed measured, both quantified in steps/minute
- Density: the ratio between the walking time and the total time elapsed in training, both quantified in minutes
- Volume: the total number of steps performed during each training session

An example of TRIMP quantification for a single exercise session is reported in Table 1.

### 2.5. Definition of the Factors of the Model

Briefly, as reported elsewhere [5], each subject under training is represented as a system with a daily exercise quantity w(t) as input and the predictive performance p(t) as output, both of which are functions of time (t). The effect, or the time impulse response, is obtained by a mathematical formula of a first-order system and is g(t) = k ∙ e^t/s^. where “k” is a magnitude factor corresponding to the increment in fatigue and/or fitness provided by every exercise session and “s” is a decay time constant, or the time necessary to observe a decrease to 37% of the initial value [5].

### 2.6. Model Development

The model, applied specifically and individually to each patient for the entire duration of the rehabilitation program, was calibrated on the actual measured performance p(m) by calculating the differences between theoretical performance p(t) and p(m) fitting those differences by the least square methods by minimizing the residual sum of squares (RSS) [10,30].

### 2.7. Statistical Analysis

Data are expressed as the mean ± standard deviation for continuous variables and number and percentage for categorical variables. A comparison between baseline and end-of-treatment PFWD and 6MWD was performed via paired-samples *t*-tests. The model of performance was calculated for each subject by hypothesizing an adaptive response to the training load and fitted for the parameters measured: PWFD and 6MWD.

The coefficient of determination R^2^ between p(t) and p(m) was calculated. The statistical significance of the fit was tested by an analysis of variance (ANOVA) on the RSS. Passing–Bablok regressions were employed to make a comparison between the actual and estimated performance for both PFWD and 6MWD. A *p*-value < 0.05 was considered statistically significant. Statistical analysis was performed with MedCalc^®^ Statistical Software version 20.014 (MedCalc Software Ltd., Ostend, Belgium).

## 3. Results

One hundred and twenty-four patients were addressed to the vascular rehabilitation program during the period of enrollment. Twenty-four patients were excluded from the final analyses due to dropping out for health reasons (neoplastic or cardiovascular disease *n* = 13) or personal reasons (family issues, *n* = 5) or incomplete program execution (*n* = 6). For the remaining 100 patients, the IR model was applied. The baseline characteristics of the final sample of PAD patients are reported in Table 2. For each patient, the baseline value of PFWD and 6MWD was considered as p(0), or the starting performance value.

### 3.1. Program Execution and TRIMPs Calculation

**Figure 1:**
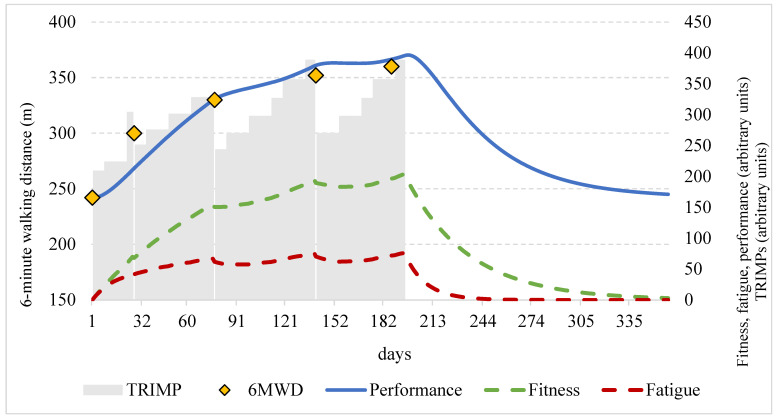
Description of the impulse–response model of a sample subject included in the study considering the 6MWD. Orange diamonds represent measured performance; blue line describes the predicted performance by IR model by subtracting positive training effects (fitness: green dashed line) to negative training effects (fatigue: red dashed line). Daily training impulses (TRIMPs) are also reported as grey columns. All variables represented as functions of time (days) in the horizontal axis.

All analyzed patients safely completed the 6-month exercise program, completing more than 90% of the scheduled training sessions for a total of 144 ± 25 km walked (during 50 ± 8 h of home training) and coming to the hospital 5 ± 1 times for check-up visits. The training load progressively increased, leading to a mean TRIMP value starting from 276 a.u. the first training day to a value of 601 a.u. the last training day after 27 ± 3 weeks. The TRIMP value progression for each training day is reported in Figure 1.

### 3.2. Performance Variation over Time and Model Fitting

Both parameters of walking ability improved over the consecutive visits, with a significant difference (*p* < 0.001) between T0 and T4 for both PFWD and 6MWD. Data are reported in Table 3.

For each patient, the actual performance was fit to the model calculated by the systems model of training with one fitness component. For PFWD and 6MWD, 89 out of 100 patients showed acceptable adherence to the estimated model (R^2^ > 0.30). The values of the constants k and τ are reported in Table 4 for both PFWD and 6MWD. The estimated decline in performance in absence of training with a return to baseline value was calculated in 18 ± 3 weeks.

The TRIMPS, estimated and actual performance for a sample subject are reported in Figure 1.

When comparing all the actual measured values with the estimated values for all time points, we observed a very high concordance, with intraclass correlation coefficient values of 0.91 (95% CI 0.88–0.92) for PFWD and 0.95 (95% CI 0.94–0.96) for 6MWD.

**Figure 2:**
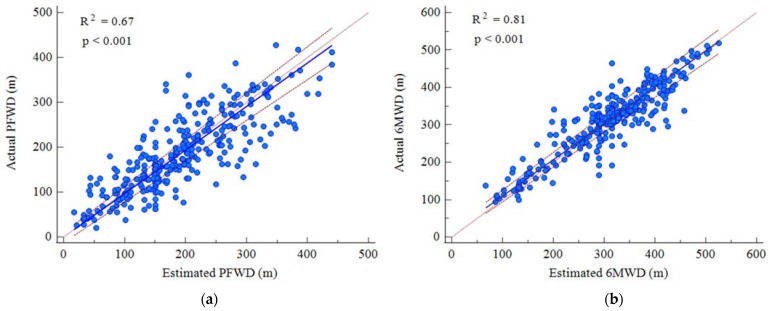
Passing–Bablok regressions between actual and estimated performance for both PFWD (**a**) and 6MWD (**b**).

The Passing–Bablok regressions for both outcomes did not show significant deviations from linearity (Figure 2).

### 3.3. Sex-Based Response of the Model

The model parameters, subsequently analyzed for the group of men (*n* = 75), and women (*n* = 25), did not reveal any significant differences in model fittings (PFWD: Men R^2^ = 0.69; Women R^2^ = 0.66; 6MWD: Men R^2^ = 0.80; Women R^2^ = 0.83). In addition, no differences in constant values were observed for the two sexes (Table 5).

## 4. Discussion

The study has shown that the changes in walking performance observed during a structured progressive pain-free exercise program correspond to those predicted by a mathematical IR model based on two components, fitness and fatigue, directly related to the training load imposed.

The study demonstrates that in a population of PAD patients an original organization of the training load based on daily short walking units at increasing speed is effective in producing predictable adaptive walking improvements even in a complex model of performance such as PAD, where multiple factors may cause a wide-ranging response. The study also shows that the model is equally valid for male and female patients of the population under study, without any difference.

We previously observed a discrete repetitiveness of the functional response to progressive training based on weekly cycles and on mesocycles of approximately 5–8 weeks [24,25,26] in PAD patients enrolled in the TiTo program [28,31,32,33,34]. This fact led us to isolate a period of enrolment to mathematically analyze the training effects on patients by an “impulse-response” model [5], where the performance is calculated integrating fitness and fatigue according to a linear response to the training load [14]. Even if modified more complex models enable higher workloads to be evaluated [8,9,30], we opted for the simple model for ease of design and for the nonmaximal load prescribed to patients. In the TiTo program, in order to obtain lower limbs aerobic adaptations in muscles exposed to an early, even not perceived, deoxygenation [35,36], the training is slower than the habitual walking speed and fragmented into short one-minute work units separated by equal rest periods.

This factor was considered in the mathematical model for the training load calculation [30], which is based on exercise units or training impulses the so-called TRIMPs [1,5,37], weighted according to their duration, frequency and relative intensity. In particular, this parameter in the present study was obtained as the ratio between the prescribed home walking speed and the habitual walking speed rather than by using heart rate, speed or lactate concentration [38,39,40,41].

The study, on the one hand, confirms the effectiveness of the exercise program in the cohort under study, with variations in 6MWD and PFWD (+33 m and +121 m, respectively) exceeding the minimal clinically important difference [42]. The relevant increase in PFWD also confirms the aerobic effects of the program, possibly supported by previously reported hemodynamic adaptations [26,28,43]. On the other hand, as the main result for the present study, the analyses show the correspondence between the changes in functional capacity calculated by the IR model as a result of the structured progressive training load and those obtained in vivo in patients enrolled in the TiTo program. This fact is relevant considering the challenging model of performance represented by PAD patients, where the number and type of lesions, different previous treatments, comorbidities and degree of deconditioning might largely affect the rehabilitative outcomes. Moreover, in addition to the general correspondence with the cohort of patients as a whole, 89 patients out of 100 showed a good mathematical correspondence with the model in terms of individual progression of the training load, and the associated effects also in the presence of possible temporary deviations from the prescription due to intercurrent illnesses or personal issues.

The study by the IR model also describes how the changes of performance (6MWD and PFWD) starting from the initial performance p(0) occurred throughout the program, according to Fitness and Fatigue response components, and at which rate the performance may decline in case of exercise interruption. Four coefficients define the magnitude of the response in terms of fitness (k_1_) and fatigue (k_2_) and their time of decay (τ_1_, τ_2_). In the present study, the τ_1_ values were comparable to those previously reported during training in healthy sedentary subjects or athletes [1,8,30], suggesting that the proposed exercise represents an effective training stimulus in subjects with reduced mobility. The τ_2_ values observed are instead higher than those reported in the mentioned studies but congruent with those calculated in a population of cardiopathic patients [12,13] for a possible slower muscle recovery of fatigue even when walking at low-moderate intensity [35].

The analysis of the coefficients also allowed to estimate the decay of performance once the training was suspended. The return to the baseline values estimated at 16–24 weeks is acceptable in terms of persistence of the benefit in absence of any stimulus, but it also confirms the need to maintain an adequate dose of exercise that is actually prescribed at the exit of the program. Purposely, the model can now allow us to calculate the lowest training load required to maintain acquired mobility, thus promoting long-term adherence to exercise.

As a final result, we studied the response according to patients’ sex. It was reported that a generic equation for both males and females [10] may affect the ability of TRIMPs to qualify the individual dose of exercise. Performance fatigability may differ between sexes for muscle characteristics [44,45] and response to physical efforts, even if some of these differences decline with age [45]. Furthermore, a different response to rehabilitation in women may derive from lower physical function and general health or psychological status at entry [46], from limited adherence to the exercise program for several barriers including the presence of pain, especially in PAD [16,17,18,47,48,49]. In this study, not only superimposable 6MWD changes were noted in the two subgroups as previously observed [50], but also in terms of coefficients defining the magnitude of fitness and fatigue and their time of decay. If the features of the in-home execution and the low intensity in the absence of pain [24,25] may bypass some of the barriers to exercise, a personalized progressive exercise may explain the comparability of the coefficients.

There are several limitations of the study. The number of serial measurements performed for each subject is less than that recommended to achieve a good level of accuracy of the model. However, the statistical results observed were acceptable for most subjects. Moreover, in a real-world situation, a high number of medical visits affects the patient’s compliance with a program [25], and purposely, the TiTo program requests few check-ups. In the TRIMP calculation, the relative intensity was calculated by a novel parameter, even with evident physiological significance in PAD [33]. The impact of pharmacological therapy changes during the study, despite in few numbers (<10%), was not investigated. Finally, the model is based only on exercise data prescribed and performed according to the diary without collecting, or measuring by wearable devices, the daily working or leisure activities which could have potentially influenced the fitness and fatigue components of the model.

## 5. Conclusions

In conclusion, in a cohort of PAD patients at intermediate stages, the changes in walking ability following structured exercise were comparable to those calculated by an IR model of performance. The study demonstrates that mathematical models originally used in sports may support a personalized, sustainable manipulation of the training variables in diseases where fatigue and pain affect adherence and rehabilitative outcomes.

## Figures and Tables

**Table 1:** Example of the calculation of the TRIMPs.

6MWT	Exercise Program			
Habitual walking speed recorded 100 steps/min.	Single training session	Walk	1 min	To be repeated 10 times
		Rest	1 min	
		Speed	60 steps/min	
Intensity = training speed ÷ walking speed = 60 ÷ 100 = 0.60 Density = walking minutes ÷ total minutes = 10 ÷ 19 = 0.53 Volume = number of steps × total repetitions = 60 × 10 = 600 TRIMP = intensity × density × volume = 0.60 × 0.53 × 600 = 191 arbitrary units				

**Table 2:** Baseline characteristics of the patients under study.

	PAD Patients *n* = 100
Males, *n*	75
Age, years	71 ± 9
Risk factors; *n*	
Smoking habit	92
Current smokers	4
Hypertension	81
Hyperlipidemia	67
Type 2 diabetes	43
Chronic kidney disease	11
Family history for cardiovascular disease	35
Comorbidities, *n*	
Ischemic heart disease	45
Stroke	15
Osteoarticular disorders	34
Pulmonary diseases	11
Neoplastic disesase	21
Charlson Comorbidity Index	3 ± 2
Age-adjusted Charlson Index	6 ± 2
Peripheral vascular disease	
Rutherford stage 1	4
Rutherford stage 2	79
Rutherford stage 3	17
Revascularizations	28
Disease duration, years	6 ± 5
Bilateral disease	75
Ankle-brachial index more impaired limb	0.59 ± 0.19
Ankle-brachial index less impaired limb	0.82 ± 0.17
Pain-free walking distance (m)	114 ± 61
6-min walking distance (m)	287 ± 85

**Table 3:** Actual and estimated values of performance over the four time periods.

	T0	T1	T2	T3	T4
PFWD (m), actual	114 ± 61	136 ± 68	178 ± 82	207 ± 80	235 ± 91
PFWD (m), estimated	-	151 ± 70	184 ± 85	205 ± 84	220 ± 94
6MWD (m), actual	287 ± 85	290 ± 83	316 ± 90	315 ± 88	320 ± 97
6MWD (m), estimated	-	286 ± 82	306 ± 82	325 ± 86	330 ± 91

**Table 4:** Model parameters including baseline performance (p(0)) and constants.

	PWFD (m)	6MWD (m)
*p* (0)	114 ± 61	287 ± 85
k_1_ (a.u.)	0.03 ± 0.06	0.02 ± 0.03
k_2_ (a.u.)	0.03 ± 0.06	0.03 ± 0.03
τ_1_ (days)	45 ± 13	37 ± 11
τ_2_ (days)	25 ± 10	26 ± 9

**Table 5:** Model parameters for the two parameters in men and women.

	PWFD (m)		6MWD (m)	
	Men	Women	Men	Women
k_1_ (a.u.)	0.03 ± 0.05	0.03 ± 0.06	0.02 ± 0.03	0.02 ± 0.03
k_2_ (a.u.)	0.03 ± 0.06	0.03 ± 0.05	0.03 ± 0.03	0.03 ± 0.04
τ_1_ (days)	45 ± 12	44 ± 11	36 ± 12	38 ± 13
τ_2_ (days)	24 ± 10	27 ± 9	25 ± 10	26 ± 9

## Data Availability

The dataset of the study is available upon request to Nicola Lamberti (nicola.lamberti@unife.it).
